# Migraine Disease Burden and Trends (1990–2021): A Multidimensional Comparative Analysis of China and Other G20 Countries

**DOI:** 10.1002/brb3.71071

**Published:** 2025-12-07

**Authors:** Rong Yang, Wen Chen, Mou Sun, Hao Feng, Bing Chen, Xiaoquan Luo, Zhou Li, Fei Qiao, Hui Tang, Haibo Ren

**Affiliations:** ^1^ Beijing Anzhen Nanchong Hospital of Capital Medical University & Nanchong Central Hospital Nanchong Sichuan China; ^2^ Department of Neurosurgery, Nanchong Central Hospital, The Second Clinical Medical College North Sichuan Medical College Nanchong Sichuan China; ^3^ Sichuan Clinical Research Center for Neurological Disease Nanchong Sichuan China; ^4^ Department of Ultrasound, Nanchong Central Hospital, The Second Clinical Medical College North Sichuan Medical College Nanchong Sichuan China

**Keywords:** autoregressive integrated moving average (ARIMA), Bayesian age‐period‐cohort (BAPC), disability‐adjusted life years (DALYs), disease burden, exponential smoothing (ES), GBD, migraine, sociodemographic index (SDI), years lived with disability (YLDs)

## Abstract

**Background:**

Migraine is a prevalent neurological disorder causing significant suffering and imposing a substantial burden on healthcare systems. Utilizing data from the Global Burden of Disease Study (GBD) 2021, this study comprehensively analyzes the current status, historical trends (1990–2021), and future projections of migraine burden in China and other G20 countries. The aim is to provide scientific evidence to inform evidence‐based health strategies.

**Methods:**

We analyzed GBD 2021 data to calculate migraine incidence, prevalence, disability‐adjusted life years (DALYs), and years lived with disability (YLDs) from 1990 to 2021. Statistical analyses characterized these metrics across age, sex, year, geographical region, and the sociodemographic index (SDI). Trends in China and other G20 countries were tracked. Future trends (2022–2050) were projected using exponential smoothing (ES) and autoregressive integrated moving average (ARIMA) models. The Bayesian age‐period‐cohort (BAPC) model quantified the effects of age, period, and cohort factors on incidence, elucidating long‐term burden drivers.

**Result:**

Migraine burden exhibited significant age dependency, with a bimodal age pattern. A pronounced gender disparity was evident, with females bearing a consistently higher burden across all metrics (e.g., prevalence approximately twice that of males). From 1990 to 2021, the migraine burden demonstrated a dual‐track trajectory: An overall increase in total burden accompanied by a widening gender gap. Predictive models project a concerning future: Without effective interventions, the migraine burden during 2030–2050 will feature further escalation of the gender imbalance. The BAPC model indicates a projected century‐long intensification of the migraine burden.

**Conclusions:**

The migraine disease burden possesses distinct age and sex dimensions, revealing significant disparities across SDI regions, countries, age groups, and genders. The escalating burden necessitates targeted interventions and public health initiatives, particularly in regions and populations disproportionately affected.

## Graphical abstract

Based on GBD 2021 data, this analysis of historical trends and current situation overviews the global burden of migraines from 1990 to 2021.

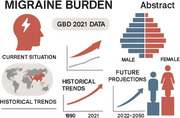

**Abbreviations**
ARIMA: AutoRegressive Integrated Moving Average
BAPC: Bayesian Age‐Period‐Cohort Model
DALYs: Disability‐adjusted life years
ES: Exponential Smoothing
GBD: Global Burden of Disease
SDI: Sociodemographic Index
YLDs: Years lived with disability

## 1 Introduction

Migraine is a common and highly disabling primary headache disorder characterized by recurrent moderate‐to‐severe, often pulsating headaches, ranking it among the leading causes of global health loss. In an authoritative database, such as the Global Burden of Disease (GBD) study, migraine is consistently positioned as a top cause of years lived with disability (YLDs), with a disability burden that exceeds the combined total of all other neurological disorders. This profound burden manifests not only at the individual level but also imposes substantial socioeconomic costs on society, encompassing significant direct medical expenses (e.g., diagnosis, treatment, medications) and even larger indirect costs stemming from reduced productivity, work absenteeism, and early retirement (Li et al. 2023; Y. Yang and Cao 2023).

The G20 comprises the world's major economies and most influential nations, representing approximately two‐thirds of the global population and over 80% of the global economic output. These countries play a pivotal role in global resource allocation, health policy formulation, and global health governance (Dieleman et al. 2019). Consequently, a thorough understanding of the current status, disparities, and determinants of the migraine burden within the G20 nations is essential for assessing its macro‐level impact, guiding the optimization of headache care systems, and informing evidence‐based interventions and resource allocation (G. Yang et al. 2013). Among the G20 members, China, as the world's most populous country, contributes significantly to the global migraine burden. Characterizing China's migraine burden profile within a comparative G20 framework is crucial not only for identifying its unique national challenges and needs but also for providing valuable insights for other emerging economies facing similar demographic transitions and healthcare system challenges. Such an analysis provides critical evidence to support targeted prevention and control policies (Xu et al. 2020).

While numerous studies have utilized GBD data to describe the global or regional burden of migraine, significant knowledge gaps remain. Prior research has often focused on descriptive analyses of single regions or has stopped short of integrating long‐term historical trends with future projections. A systematic comparison across the G20—a key policymaking bloc—that simultaneously dissects gender‐specific disparities and investigates the underlying drivers of burden, remains underdeveloped.

To address this gap, this study provides a more comprehensive and in‐depth multidimensional comparative analysis of the migraine burden. The unique contributions of this research are threefold: (1) It systematically situates China within the context of all other G20 countries, conducting both cross‐sectional and longitudinal comparisons to reveal its relative position and distinct trends within the global landscape. (2) It integrates a suite of advanced statistical models to not only describe the historical trajectory from 1990 to 2021 but also to provide robust forecasts of the future burden through 2050, with a specific focus on the projected widening of the gender gap. (3) It moves beyond descriptive epidemiology by applying decomposition analysis and Bayesian age‐period‐cohort (BAPC) modeling to deeply investigate the driving mechanisms of the migraine burden, including the effects of demographic shifts, period‐related societal changes, and generational cohort factors.

Leveraging the latest data from GBD 2021, this study systematically analyzes the epidemiological characteristics (prevalence, incidence) and disease burden (DALYs, YLDs) of migraine across G20 countries. Through this multinational comparative lens, we aim to identify similarities and differences in burden patterns, explore underlying contributing factors, and specifically examine the status, trends, and relative position of China's migraine burden within the G20. Ultimately, this work seeks to provide robust scientific evidence to support global and national strategies for migraine prevention and control.

## 2 Methods

### 2.1 Study Design and Data Sources

This study is a multidimensional comparative analysis based on publicly available data from the GBD study 2021. The GBD 2021 provides a systematic, comprehensive estimation of 371 diseases and injuries across 204 countries and territories from 1990 to 2021 (GBD 2021 Low Back Pain Collaborators 2023; GBD 2019 Diseases and Injuries Collaborators 2020). We extracted annual data on migraine incidence, prevalence, disability‐adjusted life years (DALYs), and YLDs for China and the other 18 G20 countries. Data were stratified by year, sex (male and female), and 5‐year age groups (from <5 to 95+ years). All estimates are presented with their corresponding 95% uncertainty intervals (UIs). The sociodemographic index (SDI), a composite measure of a region's development based on income, education, and fertility, was used to contextualize the burden across different development levels (GBD 2021 Low Back Pain Collaborators 2023). The data used in this study were sourced from the GBD Study 2021 database. Specific details regarding data extraction and processing, including complete query parameters and code, are stored in a publicly accessible GitHub repository. https://github.com/RongYang‐chenwen/Migraine‐Disease‐Burden.git.

### 2.2 Burden Metrics and Trend Analysis

We analyzed four key metrics: Incident cases, prevalent cases, DALYs, and YLDs. DALYs are the sum of YLDs and years of life lost (YLLs); for non‐fatal conditions like migraine, DALYs are virtually equivalent to YLDs. To facilitate comparison across populations with different age structures, we calculated age‐standardized rates (ASRs) per 100,000 population using the GBD 2021 global age standard population (Bilal et al. 2021).

To quantify the temporal trend in ASRs from 1990 to 2021, we calculated the estimated annual percentage change (EAPC). The EAPC was determined by fitting a linear regression model to the natural logarithm of the ASRs over time (*y* = *α* + *βx* + *ε*, where *y* = ln(ASR) and *x* = calendar year). The EAPC was then calculated as 100 × [exp(β) − 1], with its 95% confidence interval derived from the regression model. An EAPC > 0 indicates a rising trend, while an EAPC < 0 indicates a declining trend.

### 2.3 Decomposition Analysis of Burden Drivers

To disentangle the factors contributing to the change in the total number of migraine incident cases in China between 1990 and 2021, we employed a decomposition analysis method, adapted from the Das Gupta model. The total change in incident cases was attributed to three primary drivers: (1) Population growth, (2) population aging (changes in the age structure), and (3) epidemiological changes (changes in age‐specific incidence rates). The specific formulas used for this decomposition are provided in the Supporting Information. This analysis was performed separately for males and females to explore gender‐specific drivers.

### 2.4 Forecasting Future Trends (2022–2050)

We projected future trends in ASRs of incidence, prevalence, DALYs, and YLDs from 2022 to 2050 using two time‐series forecasting models: Exponential Smoothing (ES) and the Autoregressive Integrated Moving Average (ARIMA) model.

The optimal ES model was selected based on the Akaike Information Criterion (AIC). For the ARIMA(p,d,q) model, stationarity of the time series was first assessed using the Augmented Dickey‐Fuller (ADF) test. The parameters (p, d, q) were determined by examining the autocorrelation (ACF) and partial autocorrelation (PACF) functions, and the model with the lowest AIC was chosen.

Model Validation: To validate the predictive accuracy of our models, we performed an out‐of‐sample back‐test. Models were trained on the 1990–2010 data to forecast the known 2011–2021 period. We evaluated their performance using Mean Absolute Percentage Error (MAPE) and Root Mean Squared Error (RMSE) and benchmarked them against a simple random walk with drift model. The ARIMA model demonstrated superior predictive accuracy. The results of this validation are presented in Table S1. Projections are presented with their 95% prediction intervals (PIs) to reflect forecast uncertainty.

### 2.5 Bayesian Age‐Period‐Cohort (BAPC) Analysis

To quantify the independent effects of age, period (calendar time), and cohort (year of birth) on migraine incidence trends in China, we implemented a BAPC model. The model assumes that the number of incident cases follows a Poisson distribution. We used integrated nested Laplace approximations (INLA) for model fitting, which is a computationally efficient alternative to MCMC methods.

Given the well‐known identifiability problem in APC models (where Period = Age + Cohort), we adopted the common constraint of setting the sum of the cohort effects to zero. We used default, weakly informative priors for the hyperparameters of the random effects (age, period, and cohort) to minimize their influence on the posterior estimates. The results are presented as relative risks (RR) for each effect relative to a reference category.

### 2.6 Statistical Software and Data Availability

All data processing, statistical analyses, and visualizations were performed using R software (version 4.2.2). The forecast package was used for ARIMA and ES modeling, and the BAPC and INLA packages were used for the age‐period‐cohort analysis. A *p*‐value < 0.05 was considered statistically significant.

## 3 Results

### 3.1 China

#### 3.1.1 Baseline DiStribution Characteristics

**FIGURE 1:**
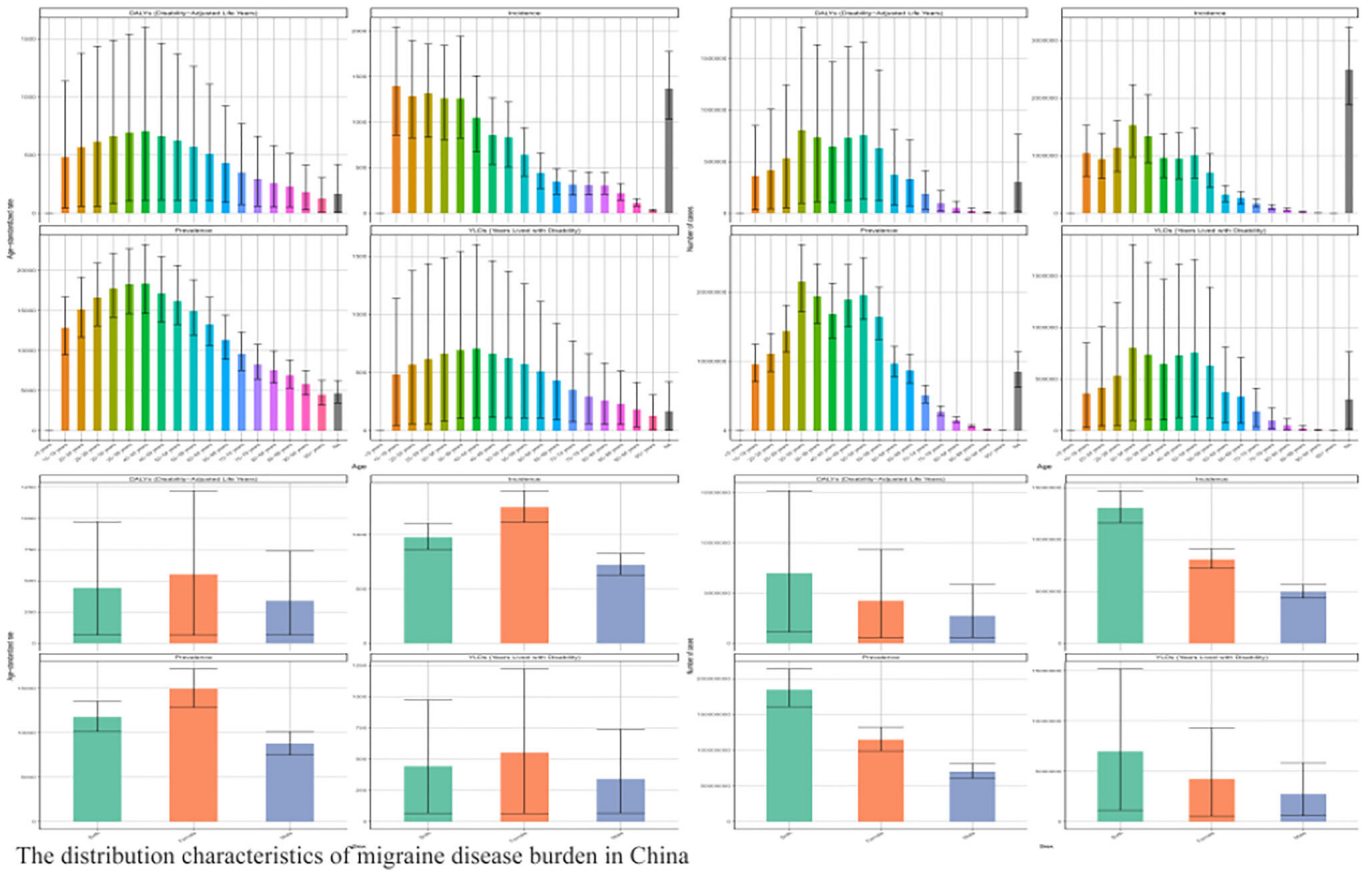
The distribution characteristics of migraine disease burden in China.

In China, the migraine burden exhibited significant age dependency in 2021. DALYs, incidence, prevalence, and YLDs all displayed a characteristic “inverted U‐shaped” age distribution, with the burden peaking between 35–55 years and the 40–49 age group bearing the heaviest absolute burden. Absolute YLDs and DALYs reached several million, quantifying a substantial disability burden. Figure 1 clearly demonstrates that YLDs constitute the vast majority of DALYs at 98.2% (95% UI: 97.5%–98.7%), indicating that the migraine burden stems primarily from long‐term disability rather than premature mortality—a core characteristic of this non‐fatal condition. The ASR component largely eliminates population age structure influence, reflecting intrinsic disease risk intensity, while the case count component illustrates the actual scale of the affected population.

**FIGURE 2:**
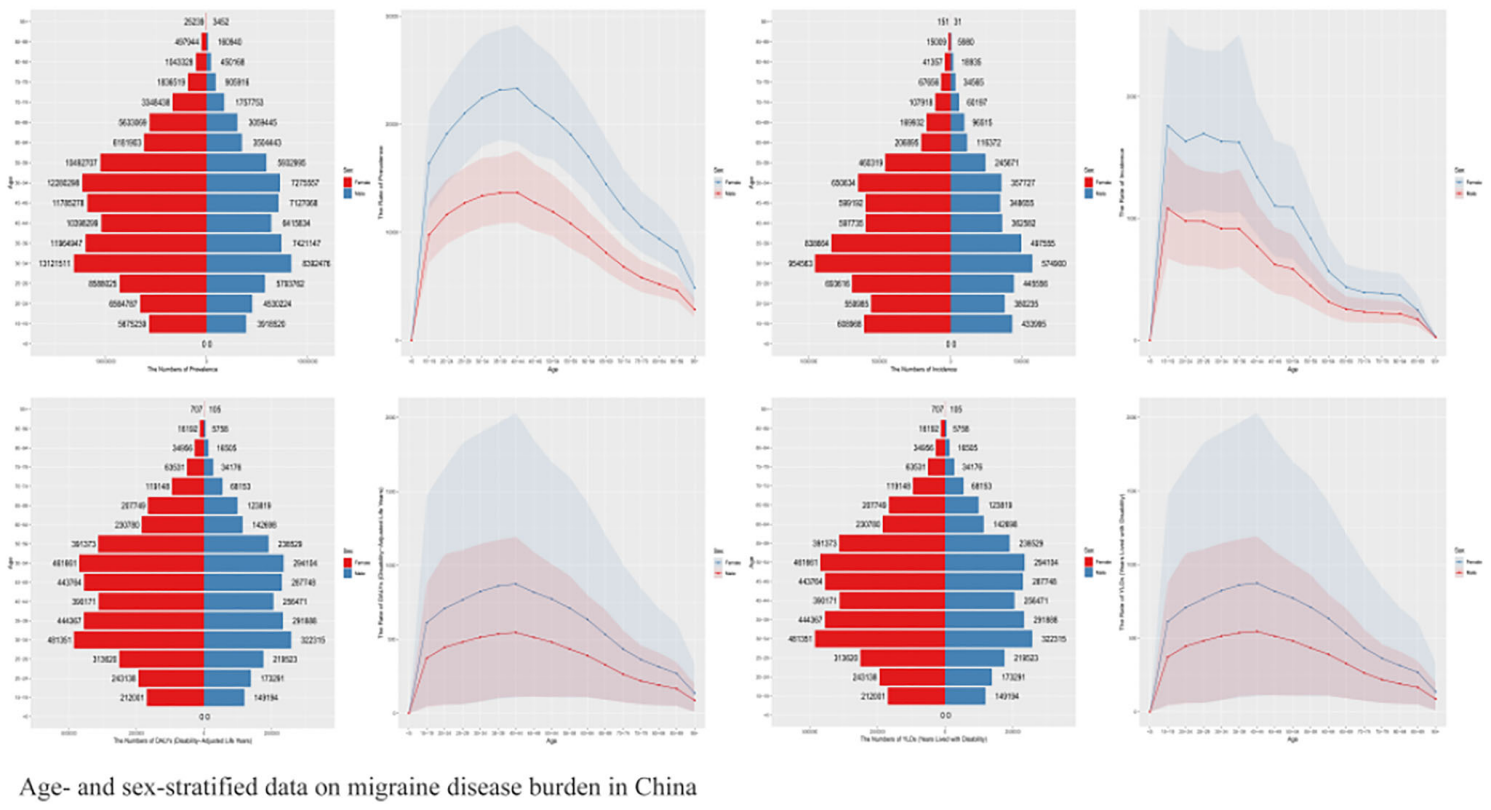
Age‐ and sex‐stratified data on migraine disease burden in China.

Migraine burden also demonstrated a significant gender disparity. Females exhibited higher values than males across all metrics, with prevalence showing the most pronounced difference. The age‐standardized prevalence rate for females was 11,850 (95% UI: 10,950–12,800) per 100,000, which was 2.2 times higher than the rate for males at 5390 (95% UI: 4880–5950) per 100,000. In absolute terms, females predominated among the approximately 145 million (95% UI: 132–159 million) prevalent cases in China for 2021. This disparity aligns with the known neuroendocrine mechanisms of migraine and female hormonal sensitivity, consistent with GBD 2019 findings (GBD 2021 Nervous System Disorders Collaborators 2024; Wang et al. 2025). Figure 2 illustrates the distinct age‐sex dual dimension: The peak burden for all metrics concentrated within the 30–49 years age group, with females experiencing a significantly higher burden than males (age‐specific prevalence was up to 1.6 times, incidence 1.7 times, and DALYs 1.5 times higher in certain age brackets). Regarding age‐specific prevalence patterns, females displayed a bimodal pattern, while males showed a more gradual increase peaking after age 50, possibly reflecting cumulative effects. At 35–39 years, female prevalent cases reached 11.96 million (95% UI: 10.8–13.1 million) (vs. 7.42 million [95% UI: 6.5–8.3 million] in males). Among incident cases aged 30–34 years, females accounted for 62.4% of the total, with 955,000 (95% UI: 850,000–1.06 million) cases out of a total 1.53 million (95% UI: 1.35–1.72 million). This disparity varied dynamically with age, being greatest during the reproductive years and persisting into older age. DALYs peaked in the 30–34 age group, with 481,000 (95% UI: 330,000–665,000) in females versus 322,000 (95% UI: 220,000–445,000) in males.

#### 3.1.2 Temporal Trajectory

**FIGURE 3:**
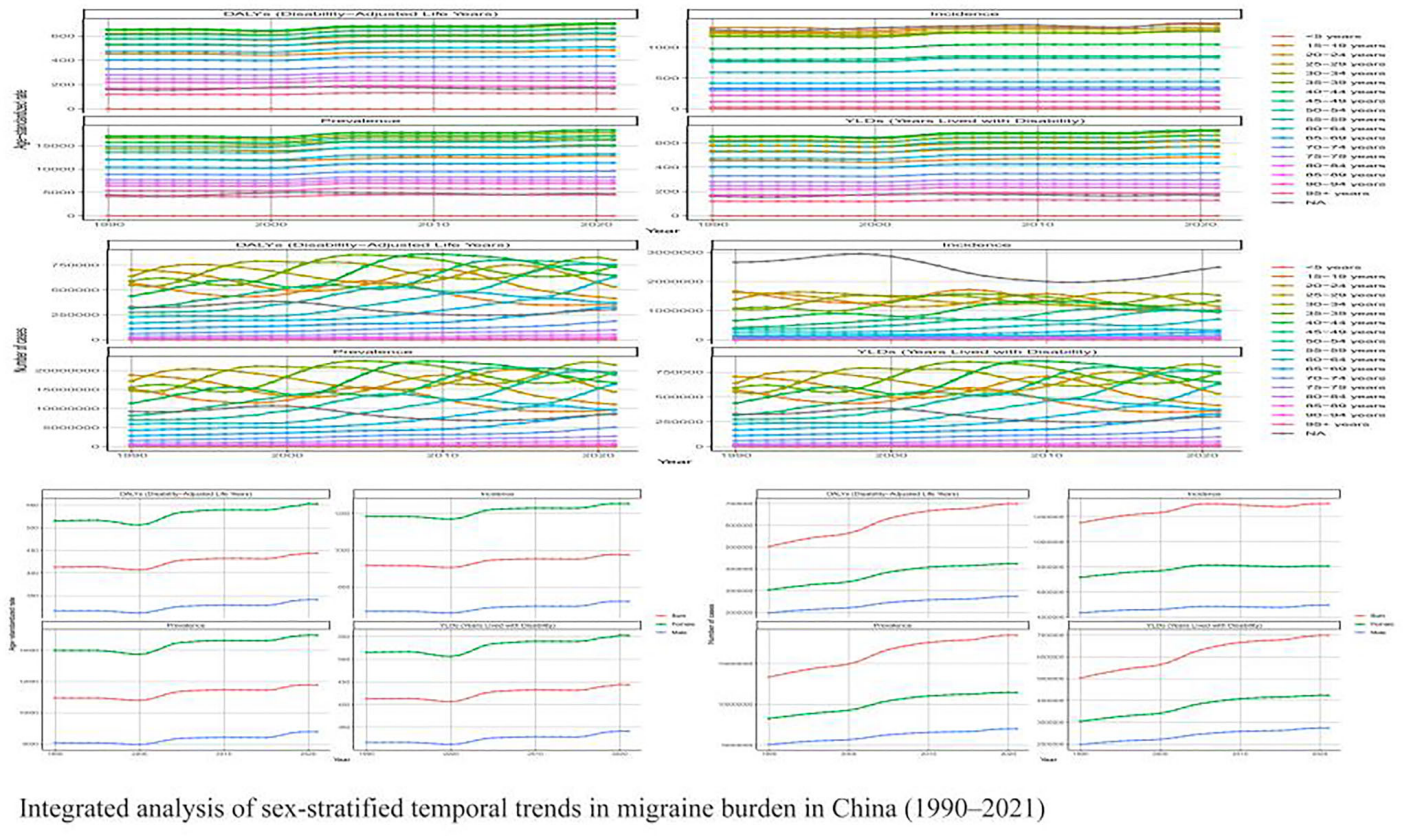
Integrated analysis of sex‐stratified temporal trends in migraine burden in China (1990–2021).

Figure 3 reveals key features in China's migraine burden trajectory from 1990 to 2021. (1) The peak burden remained confined to the working‐age population (25–54 years) across the three‐decade period. (2) A dynamically evolving gender disparity was observed: The female burden sharply surpassed that of males starting from age 15–19, with the widest gap occurring during prime reproductive years (25–39 years), where female prevalence was up to four times that of their male peers. This gap narrowed after age 75, likely attributable in part to higher male mortality. (3) A notable anomaly occurred around 2020: Synchronized increases were observed across all metrics among females aged 35–49. This was exemplified by a sharp single‐year spike in incidence for 45–49‐year‐old females that far exceeded historical trends. This observation coincides with the COVID‐19 pandemic and may suggest an exacerbating role of pandemic‐related stressors, particularly on working‐age females with parenting responsibilities, though a causal link cannot be established from this ecological data (C. Chen et al. 2025; Kalkman et al. 2023). (4) A significant increase in the burden among adolescents was noted, with incidence rates in females aged 15–19 showing a marked rise over the study period, establishing adolescence as a critical anchor point for lifelong disease burden.

**FIGURE 4:**
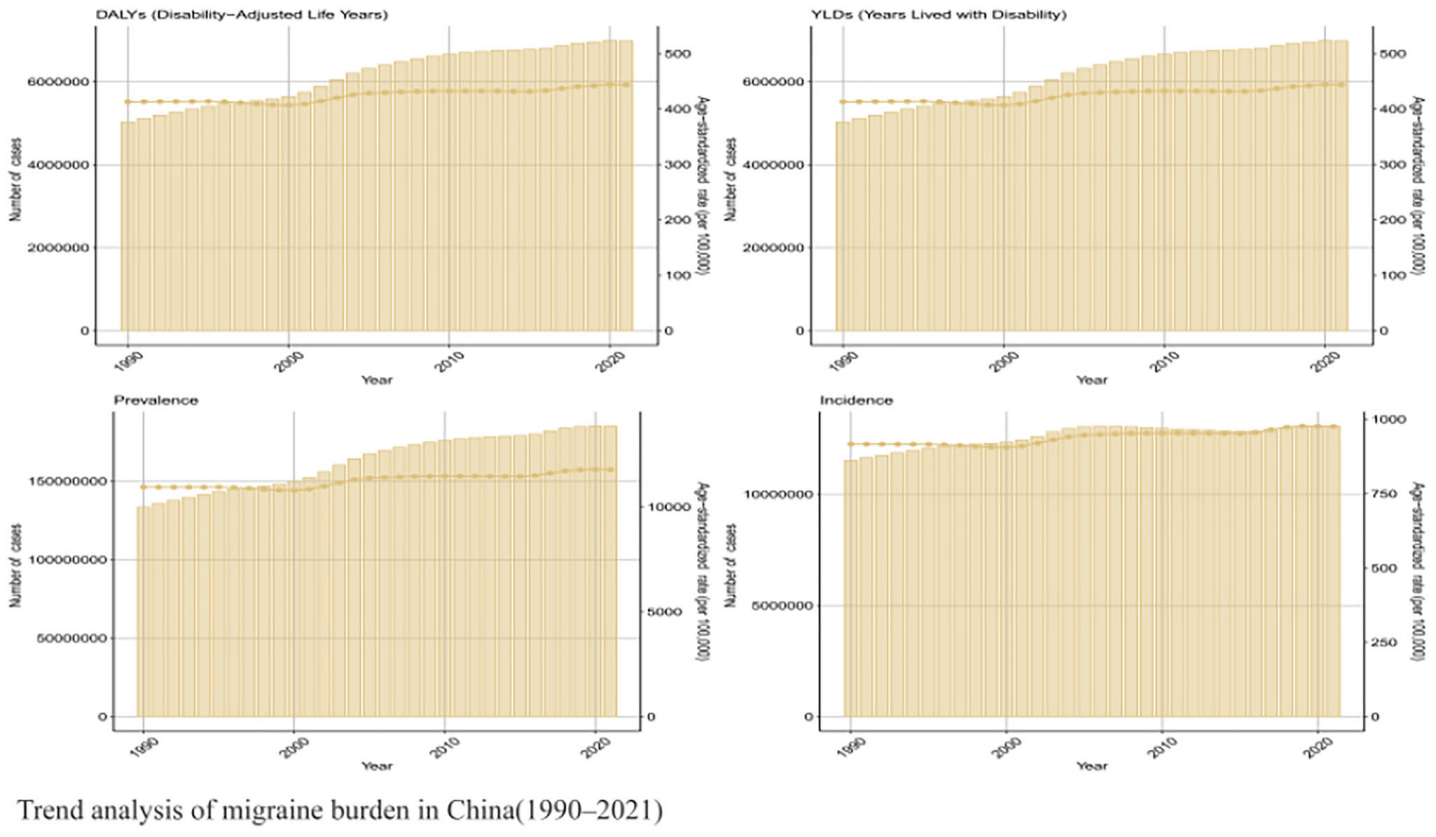
Trend analysis of migraine burden in China (1990–2021).

Figure 4 confirms a multi‐faceted increase in the burden: (1) An absolute case surge: Prevalent cases in China more than doubled, increasing by 123% from 65 million (95% UI: 58–73 million) in 1990 to 145 million (95% UI: 132–159 million) in 2021. (2) A persistent rise in age‐standardized burden: The age‐standardized prevalence rate rose by 21% (EAPC: +0.55% [95% UI: 0.48%–0.62%]) and the DALY rate by 23% (EAPC: +0.60% [95% UI: 0.51%–0.69%]) over the same period. (3) An apparent epidemiological shift: Incidence among youth cohorts increased, potentially linked to evolving societal factors such as digital stress exposure, while the disability burden in elderly groups also grew, likely driven by disease chronicity and an aging population (Kalkman et al. 2023).

#### 3.1.3 Decomposition Analysis and Forecasting

**FIGURE 5:**
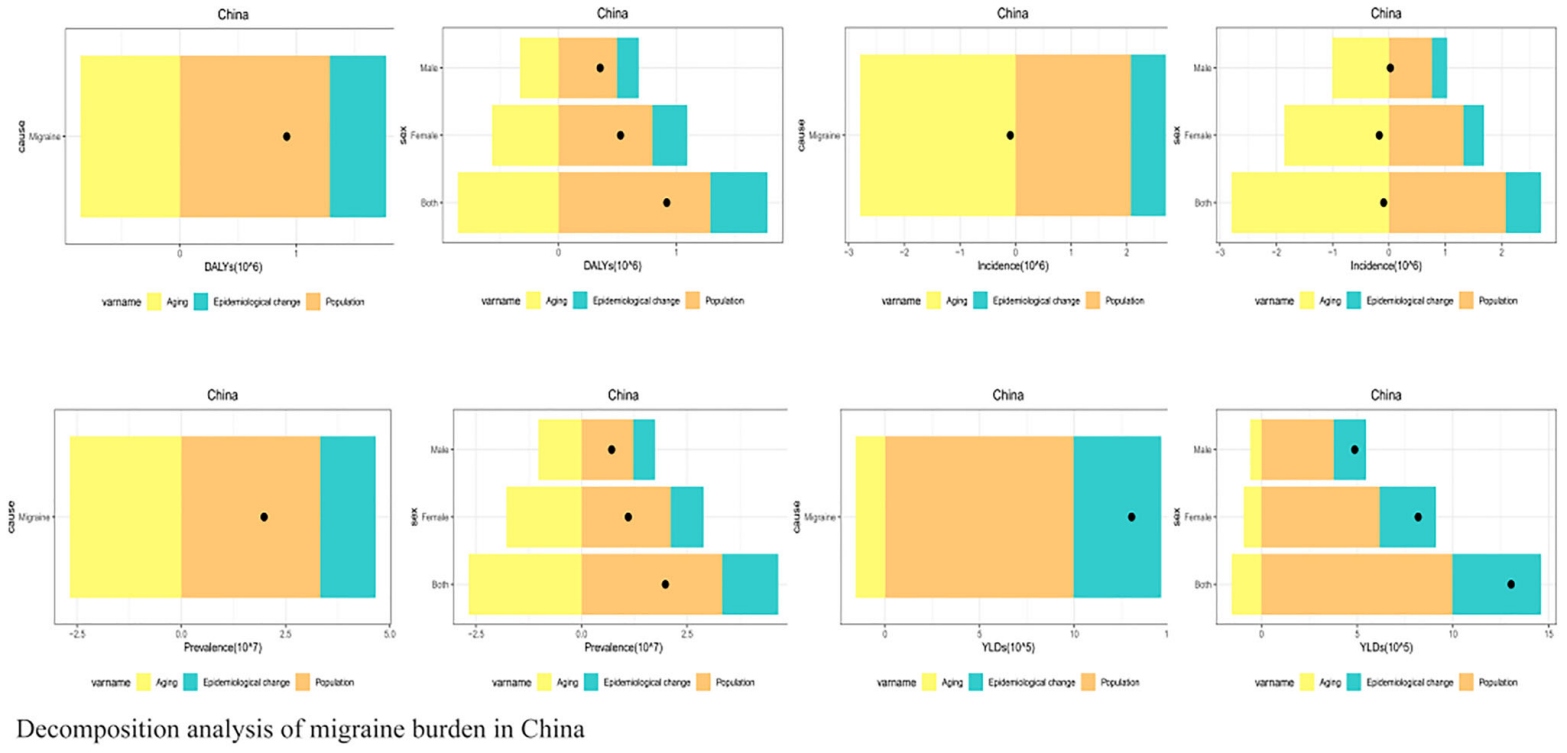
Decomposition analysis of migraine burden in China.

Decomposition analysis (Figure 5) of the increase in incident cases from 1990 to 2021 revealed a tripartite gendered structure: (1) For the increase in female cases, population aging was the primary contributor (48.2%), followed by population growth (35.5%), while epidemiological changes (i.e., changes in age‐specific rates) made a negative contribution (−13.7%). In contrast, for males, population growth was the dominant driver (55.1%) (Xie et al. 2025). (2) A disproportionate burden increase was observed among working‐age females. For instance, between 2010 and 2021, women aged 35–49 accounted for over 60% of the total increment in DALYs. (3) The “gender paradox of longevity” was evident, where longer female life expectancy extends the duration of suffering from this chronic condition, resulting in a large number of elderly women experiencing debilitating pain for over two decades (Xiao et al. 2025).

**FIGURE 6:**
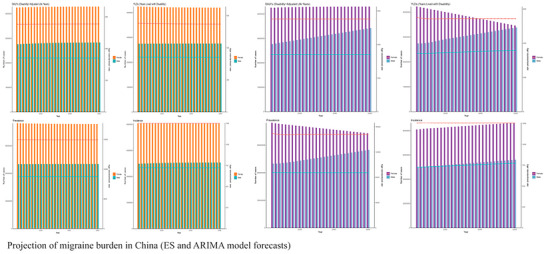
Projection of migraine burden in China (ES and ARIMA model forecasts).

Projection models, validated via back‐testing, depict a concerning trajectory for China (Figure 6; Figure S1). Without effective interventions, the gender imbalance in migraine burden is projected to intensify through 2050. Key predictions include: (1) Absolute increase: Total prevalent cases in China are projected to exceed 190 million (95% Prediction Interval, PI: 175–210 million) by 2050, an increase of 31% from 2021. (2) Deepening gender disparity: Female DALYs are projected to increase at a rate 1.8 times that of males. The female age‐standardized YLD rate is forecasted to surpass 600 per 100,000. (3) Accelerated increase in females: All female burden curves are projected to steepen during the 2030–2040 period; for example, female annual incident cases are expected to exceed 10 million by 2045. (4) Persistent working‐age burden: Working‐age women will continue to face significant pressure. By 2050, the prevalence in females aged 35–49 is forecasted to reach 18,500 per 100,000 (95% PI: 17,000–20,000), with YLD rates reaching 5200 per 100,000 (95% PI: 4500–6000). The ARIMA model suggests the gendered migraine burden may enter an accelerated growth phase post‐2030.

#### 3.1.4 BAPC Analysis for China

**FIGURE 7:**
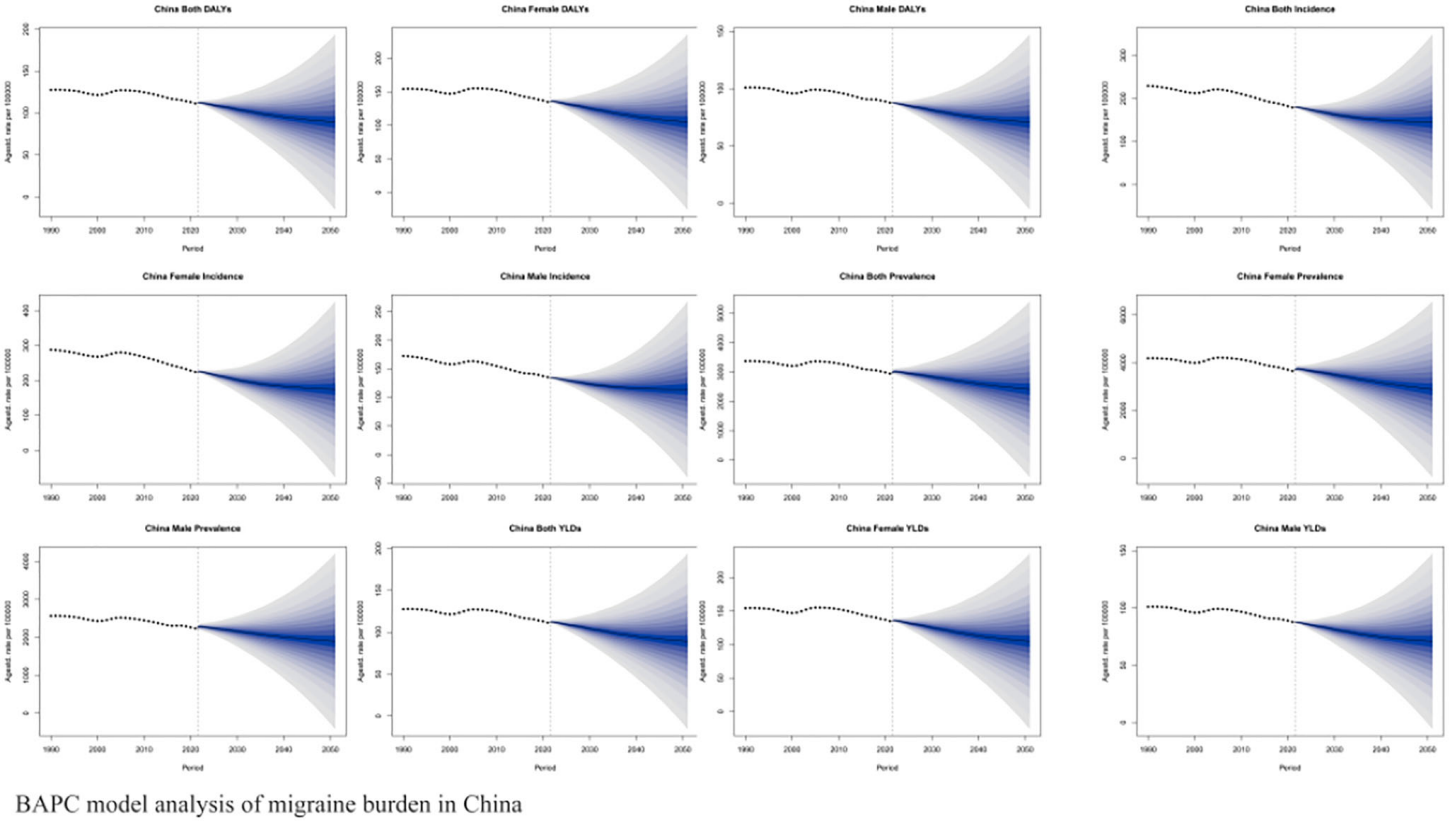
BAPC model analysis of migraine burden in China.

BAPC modeling revealed the underlying temporal drivers of incidence trends in China, providing deeper insights into the historical data (Figure 7). The analysis quantified four key features: (1) The Age effect was confirmed as the primary driver shaping the burden, with an inverted U‐shaped pattern corresponding to the age distribution of incidence. (2) The model quantified the gender disparity, with the RR from the age effect being consistently higher for females across the lifespan, a finding potentially linked to hormonal factors such as perimenopausal fluctuations (Delaruelle et al. 2018). (3) The period effect showed an inflection point, with a period of slowed growth during 2010–2020, which may reflect the impact of public health initiatives such as the nationwide implementation of the Chinese Guidelines for Migraine Prevention and Treatment. (4) The Cohort effect pointed to a future concern: Successive birth cohorts, particularly those born after 1980, exhibited a progressively higher RR of incidence compared to earlier generations. This suggests that as the population ages, it may enter a high‐risk phase for migraine burden, a trend that reinforces the concerns raised by our forecasting models (X. Chen et al. 2022).

### 3.2 G20 Countries

#### 3.2.1 Baseline Distribution Characteristics

**FIGURE 8:**
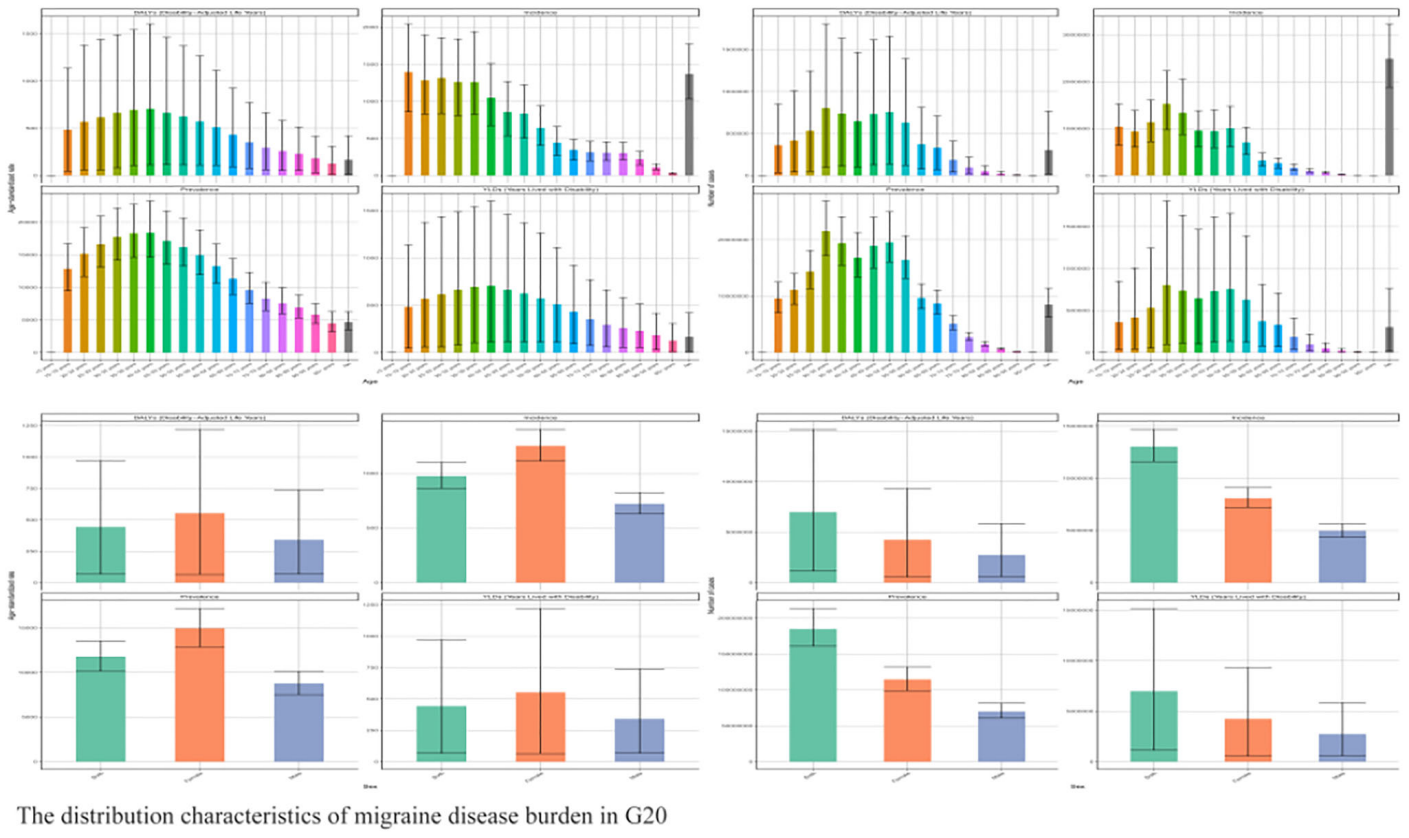
The distribution characteristics of migraine disease burden in G20.

**FIGURE 9:**
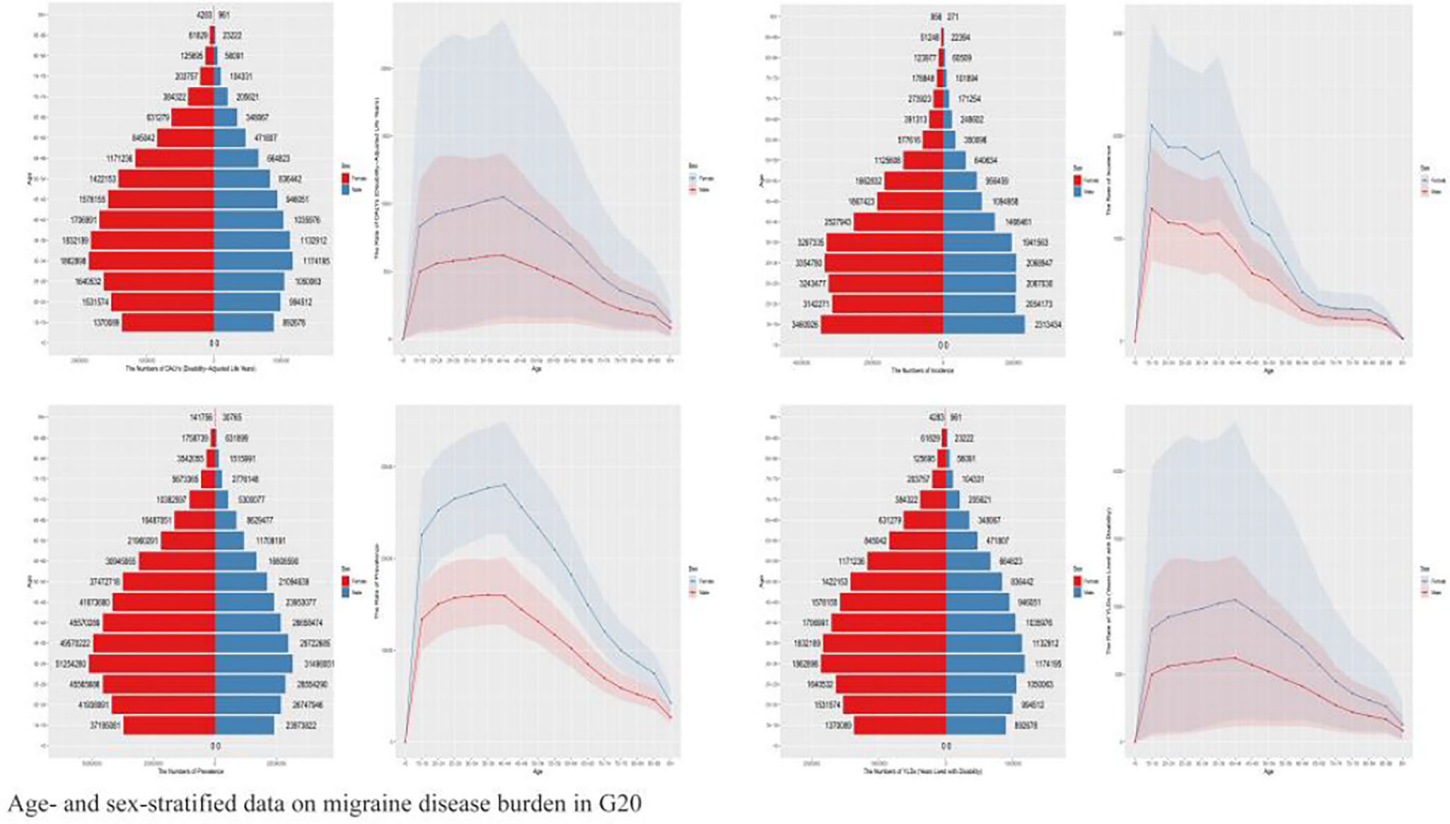
Age‐ and sex‐stratified data on migraine disease burden in G20.

To provide context for the G20 analysis, the worldwide migraine burden showed a persistent increase from 1990 to 2021. Globally, prevalent cases rose by 68% to reach an estimated 673 million (95% UI: 610–740 million) in 2021 (Figures 8 and  9). Females consistently bore a disproportionately higher burden; in 2021, global female prevalence (442 million cases) was 1.9‐fold higher than that of males (231 million cases). This pattern highlights the global scale of the issue this study addresses within the G20 (Pozo‐Rosich 2025).

Within the G20, the fundamental characteristics of the migraine burden were consistent. Age distribution analysis revealed a bimodal pattern across all metrics, with the lowest burden in children under 5, a first peak in young adulthood (25–29 years), and the highest burden concentration at 50–54 years, before a gradual decline after age 75.

A pronounced gender imbalance was a universal feature across all G20 nations. For the G20 aggregate, the age‐standardized prevalence rate for females was 14,920 (95% UI: 13,980–15,850) per 100,000, which was 2.1 times higher than the rate for males at 7210 (95% UI: 6550–7890) per 100,000. This disparity was most acute in disability metrics, where females accounted for approximately 70.2% of total DALYs. Sex‐age interaction analysis further uncovered that females experienced higher burdens at all ages, peaking during the perimenopausal period (50–54 years). Disease progression also showed sexual dimorphism, with the peak of incidence occurring approximately 5 years later in females (30–34 years) than in males (25–29 years).

**FIGURE 10:**
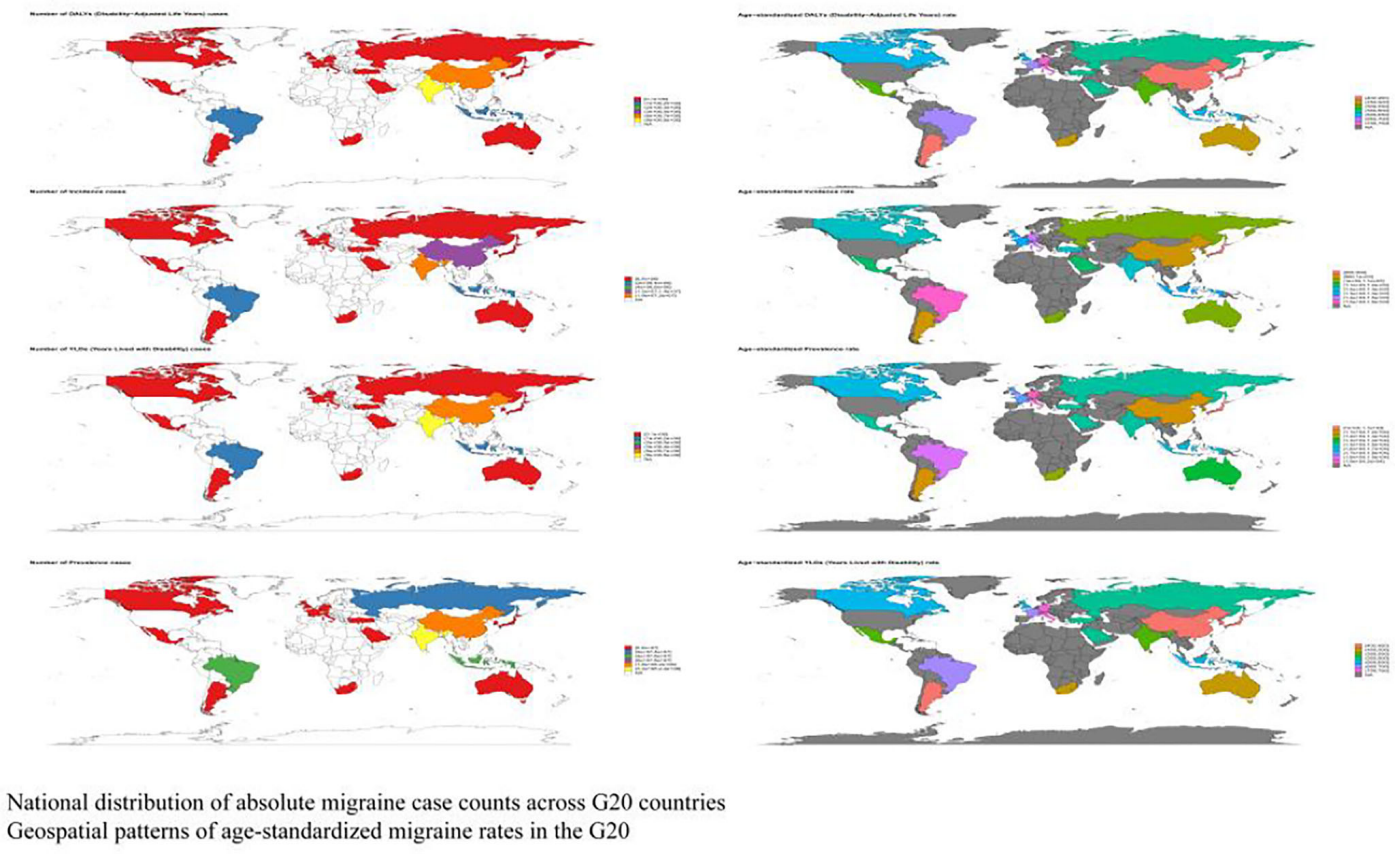
National distribution of absolute migraine case counts across G20 countries. Geospatial patterns of age‐standardized migraine rates in the G20.

Geographically, the burden varied significantly (Figure 10). ASRs were heaviest in high‐income G20 regions like Western Europe, North America, and Australasia, where prevalence rates often exceeded 15,000 per 100,000, suggesting potential influences of genetic or environmental factors (Cen et al. 2024). In terms of absolute numbers, Asian G20 countries dominated due to their demographic weight. China (145 million cases) and India (138 million cases) together shouldered a substantial burden, collectively accounting for 58.3% of the 485 million (95% UI: 440–530 million) total prevalent cases in the G20. As a group, the G20 nations bore a significant portion of the worldwide burden, representing 72.1% (95% UI: 70.5%–73.8%) of the global prevalent cases in 2021.

**FIGURE 11:**
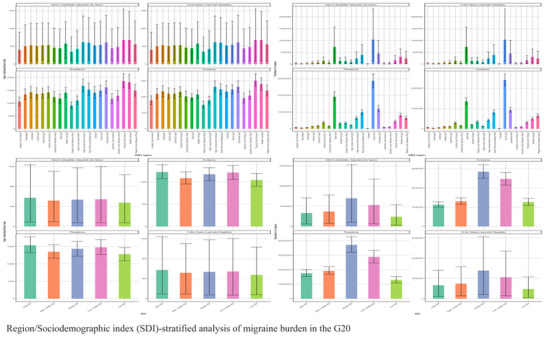
Region/Sociodemographic index (SDI)‐stratified analysis of migraine burden in the G20.

In terms of absolute numbers, China and India shouldered substantial burdens, collectively accounting for 58.3% of the 485 million (95% UI: 440–530 million) total prevalent cases in the G20 for 2021 (Figure 11). The US and Indonesia followed in case volume, while Saudi Arabia and Australia had the lightest burdens, each with fewer than 5 million cases. Analysis of epidemiological profiles revealed distinct patterns: High‐income countries (e.g., US, Japan) typically showed “high prevalence‐moderate incidence” patterns, suggesting an accumulation of chronic cases. In contrast, some middle‐income nations (e.g., Brazil, Indonesia) had more comparable prevalence and incidence rates, potentially reflecting challenges in healthcare access or treatment retention. The G20 nations as a group bore a significant portion of the worldwide burden, representing 72.1% (95% UI: 70.5%–73.8%) of the global prevalent cases in 2021.

Age‐standardized burden intensity, which reflects the per capita burden, also varied markedly. European G20 countries exhibited some of the highest rates; for example, Germany's age‐standardized prevalence was 15,800 (95% UI: 14,500–17,100) per 100,000, and Italy had one of the highest YLD rates at 750 (95% UI: 510–1,050) per 100,000. The US followed with a high prevalence of 14,800 (95% UI: 13,500–16,100) per 100,000. As noted previously, China showed a unique “high incidence‐moderate prevalence” profile (age‐standardized incidence: 3450 [95% UI: 2980–3950]; prevalence: 9560 [95% UI: 8770–10,410] per 100,000), potentially indicating gaps in primary prevention or the transition to chronic care. Saudi Arabia had the lowest metrics, with a prevalence below 6000 per 100,000, which may be constrained by factors such as disease awareness and medical coverage. Notably, the burden in Russia and Turkey exceeded levels that would be predicted by their SDI alone, suggesting a need for targeted health system optimization.

**FIGURE 12:**
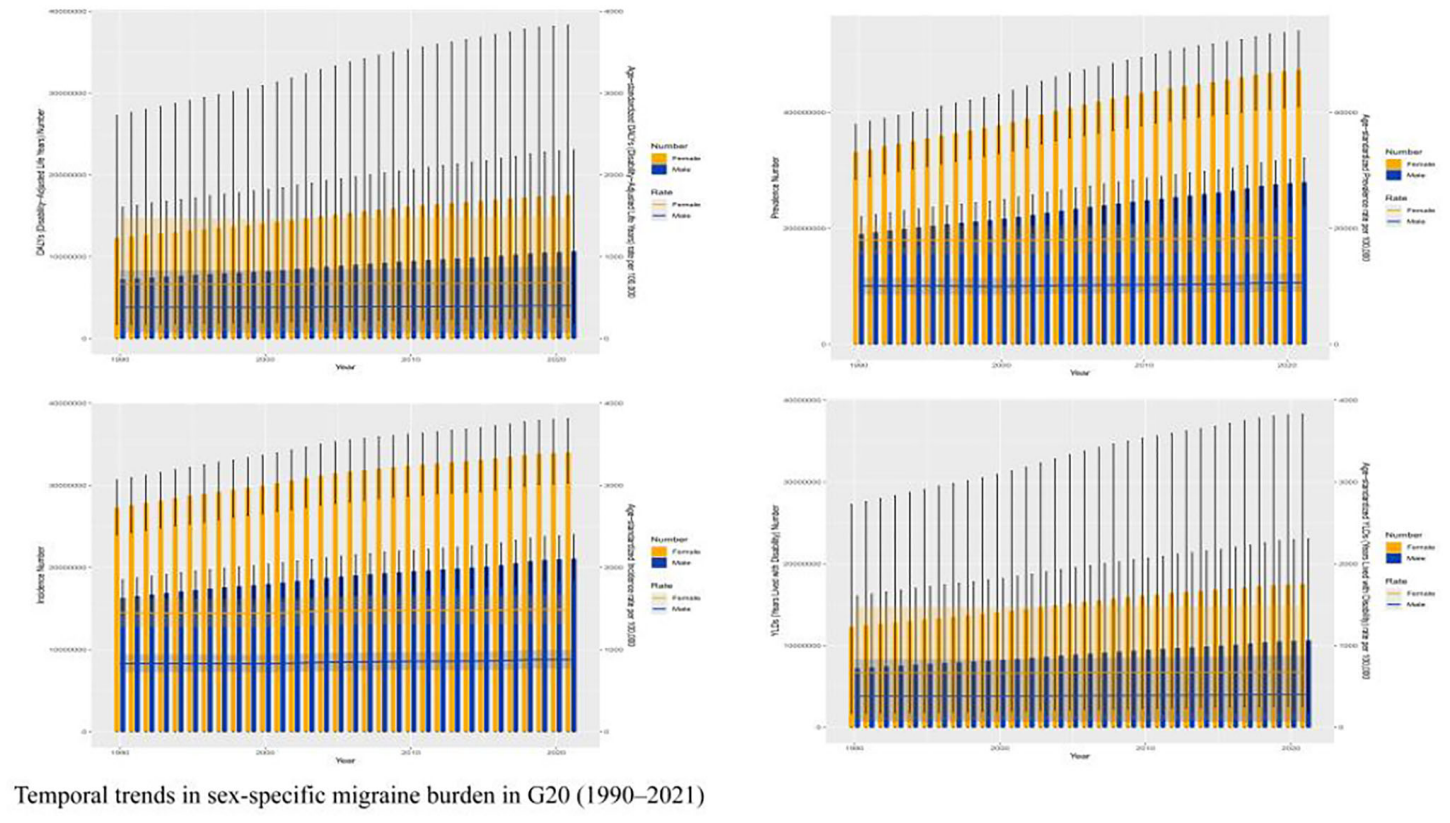
Temporal trends in sex‐specific migraine burden in G20 (1990–2021).

The 1990–2021 period witnessed dual‐track trends for the G20—aggregate growth in absolute numbers and a widening gender gap in standardized rates (Figure 12). Absolute prevalent cases in the G20 increased by 72% from 282 million (95% UI: 255–310 million) in 1990 to 485 million (95% UI: 440–530 million) in 2021, primarily due to population growth. However, ASRs revealed a sustained deterioration of the female burden. The female age‐standardized prevalence rate increased significantly from 13,950 (95% UI: 12,980–14,950) per 100,000 in 1990 to 14,920 (95% UI: 13,980–15,850) per 100,000 in 2021 (EAPC: +0.28% [95% UI: +0.21% to +0.35%]). In contrast, the male burden stagnated over the same period (EAPC: +0.05% [95% UI: −0.02% to +0.12%]). An accelerated increase in female rates post‐2010 was correlated with evolving societal pressures and improved diagnosis. A transient anomaly was also observed in 2020, where the male age‐standardized DALY rate showed a slight decline, which may reflect a temporary impact of the COVID‐19 pandemic on healthcare‐seeking behaviors or data reporting (C. Chen et al. 2025).

#### 3.2.2 Temporal Trends

**FIGURE 13:**
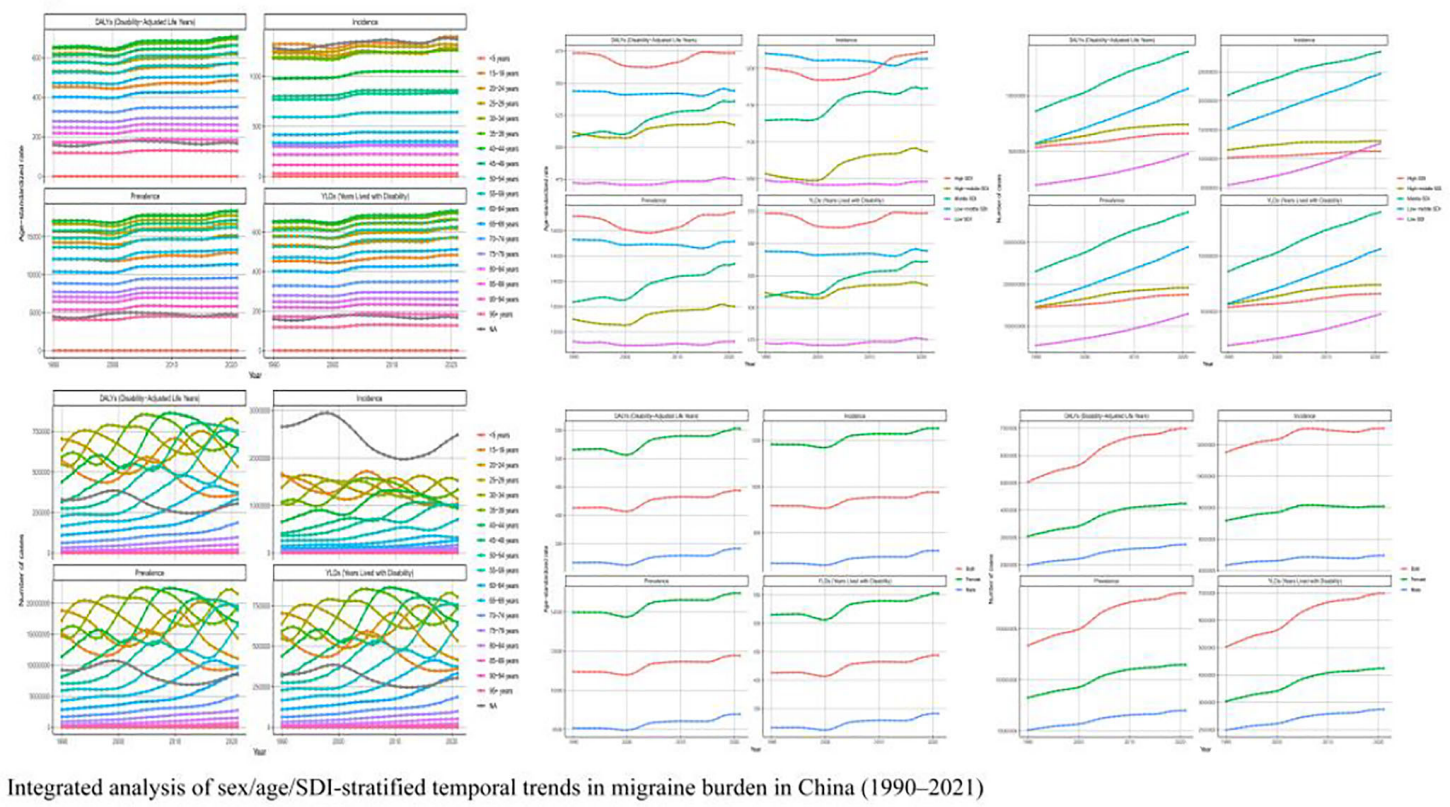
Integrated analysis of sex/age/SDI‐stratified temporal trends in migraine burden in the G20 (1990–2021).

The 1990–2021 period witnessed complex and divergent temporal trends in the migraine burden across the G20. While the aggregate burden surged in absolute terms, the patterns of change varied significantly by age, sex, and a country's development status (Figure 13).

Age‐Divergent Trends: An analysis of age‐specific trends revealed a divergence between younger and older populations. A notable increased burden was observed in young and middle‐aged adults (30–54 years). For instance, in the 50–54 age group, the absolute number of prevalent cases grew substantially, and DALY rates increased, potentially reflecting the cumulative impacts of factors like workplace stress and perimenopausal health changes. This age group emerged as an epicenter for the absolute burden increase. Conversely, the group aged over 75 years showed a relative improvement in burden, with declining age‐specific YLD rates over the three decades, a trend that may be partly attributable to advances in pain management for the elderly. A noteworthy finding was that while incidence rates remained stable in the 25–29 age group, the age‐specific YLD rates for females in this cohort increased, indicating a potential worsening of disability severity among young women.

Female‐Dominated Progression and Accelerating Gender Disparity: As highlighted previously, the progression of the migraine burden was female‐dominated. The absolute growth in cases was disproportionately female (a 78% increase in female cases vs. a 63% increase in male cases from 1990 to 2021), with females comprising 68.2% of total prevalent cases in the G20 by 2021. The acceleration in female ASRs post‐2010 was correlated with evolving societal pressures and improved diagnostic practices.

SDI‐Dependent Divergence: The evolution of the burden also showed a clear divergence based on the SDI. High‐SDI G20 nations saw modest but significant increases in their ASRs (e.g., YLDs EAPC: +0.35% [95% UI: +0.28% to +0.42%]), while their absolute case numbers grew more slowly. This pattern suggests an accumulation of chronic cases and enhanced diagnostic refinement over time. In contrast, middle‐SDI nations, including emerging economies like China and India, absorbed the largest share of the absolute growth. Their prevalent cases increased substantially, from approximately 150 million in 1990 to 355 million in 2021, constituting the majority of the G20's total burden increment. This surge, driven primarily by population growth, resulted in their ASRs remaining stable or slightly decreasing. The burden in low‐SDI G20 countries remained the lowest, although this could reflect potential underestimation due to diagnostic and reporting gaps.

**FIGURE 14:**
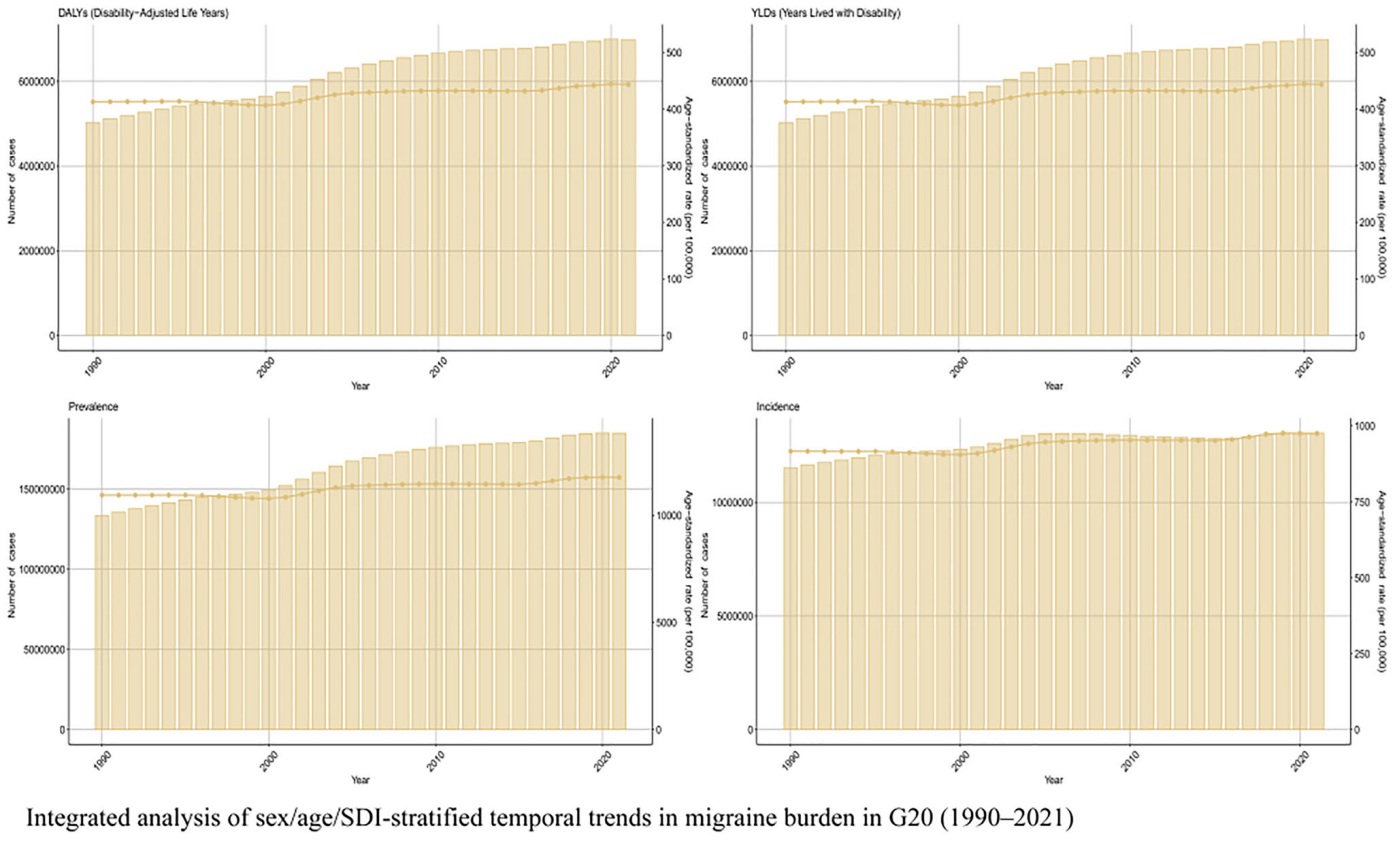
Trend analysis of migraine burden in the G20 (1990–2021).

Aggregate Burden Changes: In aggregate, the G20 migraine burden underwent significant changes, characterized by an absolute surge coupled with an intensification of disability (Figure 14). Total prevalent cases rose by 72% from 282 million (95% UI: 255–310 million) in 1990 to 485 million (95% UI: 440–530 million) in 2021, a change primarily driven by population growth and aging. Analysis of standardized rates revealed a worsening of disability severity, as the age‐standardized YLD rate for the G20 aggregate showed a significant increase (EAPC: +0.25% [95% UI: +0.18% to +0.32%]). The overall age‐standardized DALY rate grew more slowly, reflecting the offsetting effect of reduced premature mortality from other causes in the general population. The G20 nations contributed to approximately 75% of the global growth in prevalent cases from 1990 to 2021, with China and India together accounting for over 60% of the G20's case increase, confirming the core role of emerging economies in the shifting global landscape of migraine (Wu et al. 2025).

**FIGURE 15:**
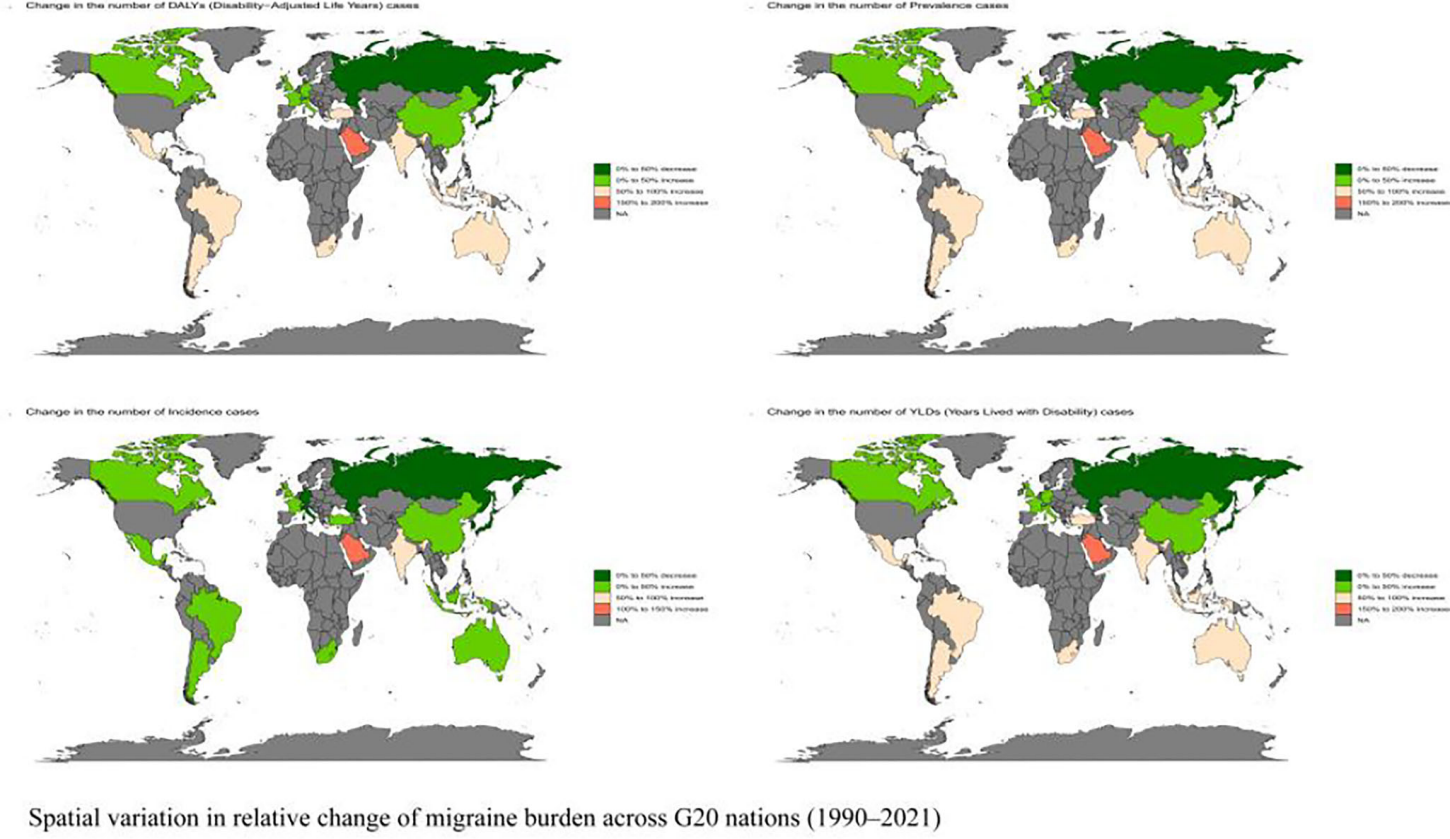
Spatial variation in relative change of migraine burden across G20 nations (1990–2021).

A distinct pattern of growth emerged across the G20, characterized by substantial absolute increases in major emerging economies versus more modest rises in high‐income nations (Figure 15). China and India drove this trend: Between 1990 and 2021, prevalent cases in China increased by 123% (95% UI: 110%–135%) and in India by 115% (95% UI: 102%–128%). Together, these two nations accounted for over 60% of the total increment in prevalent cases across the G20. Other emerging G20 economies, such as Indonesia and Brazil, also saw substantial rises in absolute cases, a trend that may be associated with demographic shifts and evolving lifestyle factors during industrialization. Conversely, most high‐income nations showed more muted growth; for example, prevalent cases in Japan increased by 38% (95% UI: 31%–46%). Saudi Arabia was a notable outlier, being the only G20 country to record a negative EAPC for its age‐standardized incidence rate. Furthermore, some countries presented unique trajectories, such as Russia's relatively low increase in YLDs compared to its overall burden, which, along with known data gaps in other regions, may indicate potential variations in healthcare surveillance and reporting systems.

**FIGURE 16:**
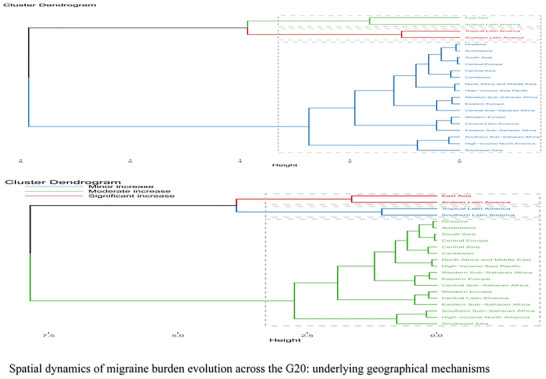
Spatial dynamics of migraine burden evolution across the G20: Underlying geographical mechanisms.

An exploratory grouping of G20 countries based on their burden trajectories from 1990 to 2021 revealed several illustrative patterns (Figure 16). One group, which included nations like China and Argentina, was characterized by significant increases in absolute burden, potentially reflecting shared demographic pressures and evolving diagnostic landscapes. Another group, comprising countries like India and Australia, showed a more moderate increase. A third distinct group consisted of high‐income nations such as the US, Japan, and Germany. This group could be described as a “high chronic disability” cluster, exhibiting relatively minor increases in ASRs over the period but consistently maintaining the highest per capita disability intensity within the G20.

#### 3.2.3 Decomposition Analysis of Burden Drivers in the G20

**FIGURE 17:**
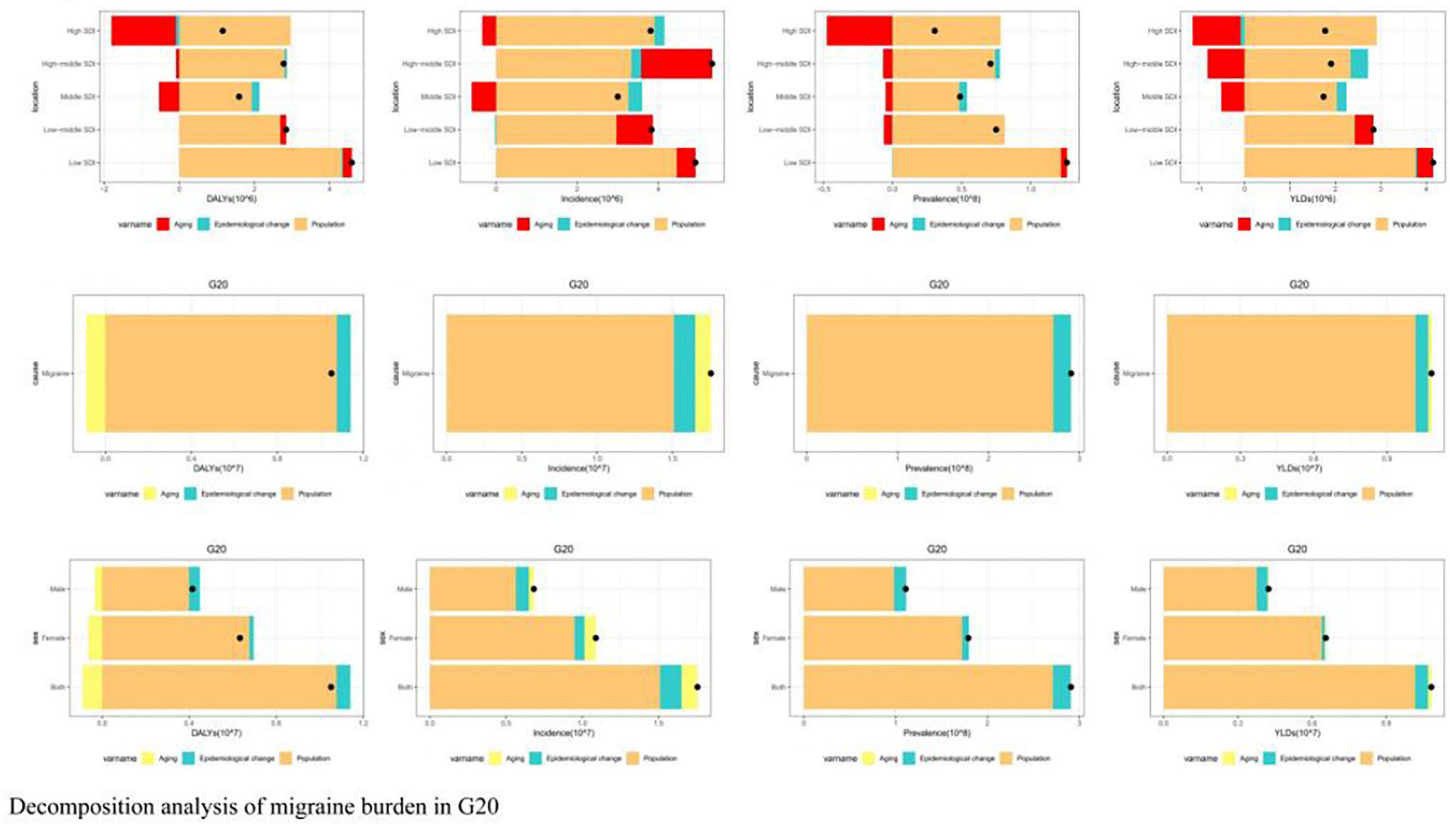
Decomposition analysis of migraine burden in G20.

Decomposition analysis of the change in incident cases from 1990 to 2021 uncovered significant gradients in migraine burden drivers that correlated with development level (Ge and Chang 2023) (Figure 17). Among G20 nations, a distinct pattern emerged: In high‐income countries such as the US, Germany, and Japan, population aging was the dominant driver, contributing to over 70% of the increase in DALYs. This reflects an “aging‐dominated disability crisis” in these nations. In contrast, in middle‐income nations like India and Brazil, population growth was the primary factor, contributing to over 60% of the increase in incident cases, highlighting the pressures of demographic expansion.

As previously detailed for China, the country demonstrated a “dual‐driver” profile where both population growth and aging jointly elevated the burden. However, China stood out by achieving a negative contribution from epidemiological changes, a favorable trend that coincided with a period of significant primary care reforms and the implementation of national migraine guidelines.

Gender stratification of the decomposition analysis for the G20 aggregate revealed that the rising female burden was primarily driven by population aging (contributing approximately 45% to the DALY increase), whereas the male burden was more dependent on population growth (contributing approximately 55% to the incidence increase). This finding underscores the necessity of sex‐differentiated prevention strategies to address the distinct drivers affecting men and women.

**FIGURE 18:**
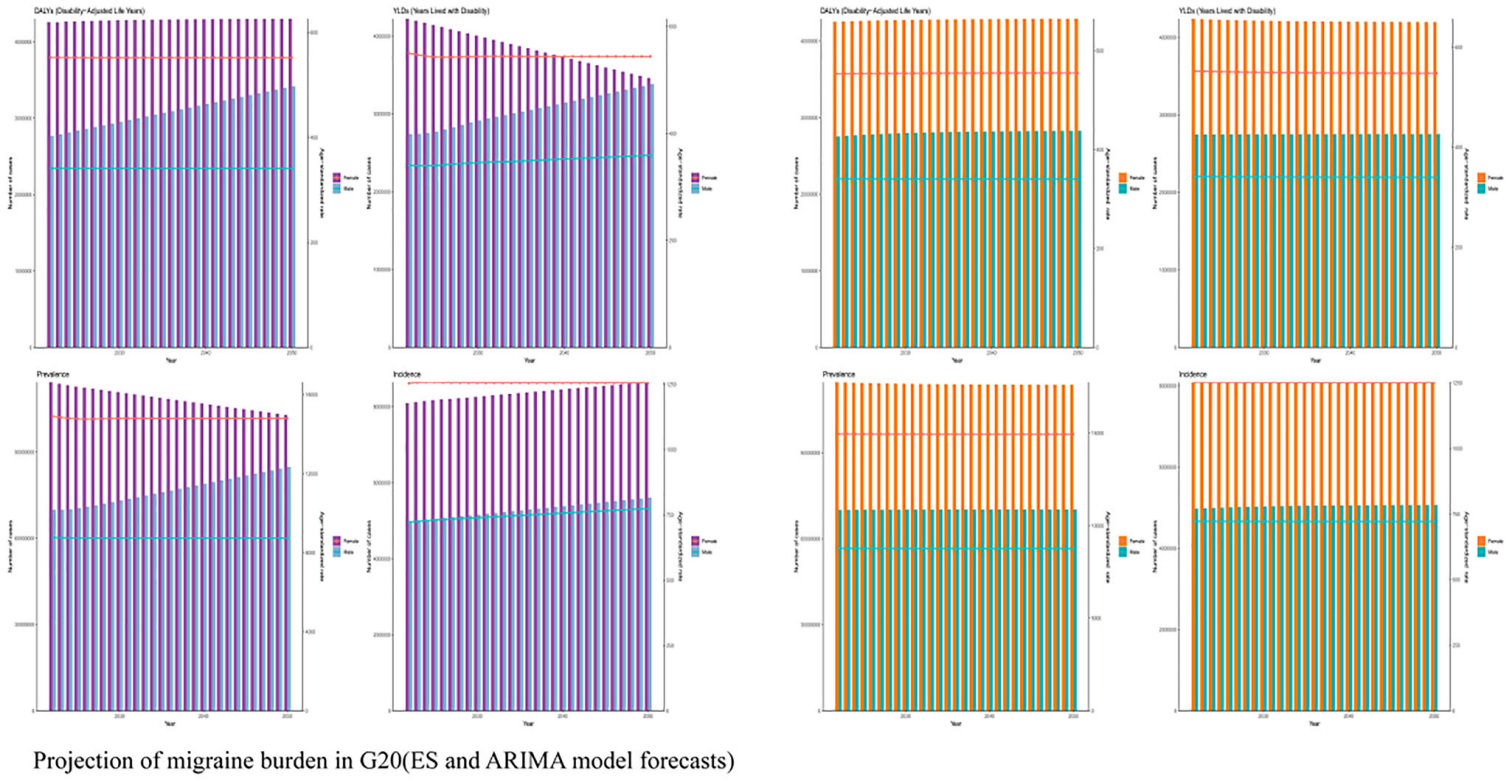
Projection of migraine burden in G20 (ES and ARIMA model forecasts).

Projections based on our validated time‐series models indicate a substantial and sustained increase in the migraine burden across the G20 through 2050 (Figure 18). Total prevalent cases in the G20 are forecasted to increase by 40%, reaching 680 million (95% prediction interval, PI: 610–760 million) by 2050.

Gender inequality is projected to intensify. The absolute number of female DALYs is forecasted to be substantially higher than that of males by 2050, with the gap between them continuing to widen. Working‐age women (35–54 years) are expected to continue facing a high disability burden, with projected age‐specific YLD rates approaching 800 per 100,000 in high‐burden countries. The overall age‐standardized YLD rate for the G20 is projected to rise, signaling a trend towards chronicity progression and a deepening of the disability burden. G20‐specific projections suggest that China and India will continue to bear the largest share of the absolute burden, while high‐income countries like the US and Japan will confront significant challenges related to the growing disability among their aging female populations.

The ARIMA model, which often captures more complex temporal dynamics, predicts a potential acceleration of these trends. It is hypothesized that a confluence of factors could influence the trajectory of the migraine burden post‐2035, including potential amplification of attack frequency associated with climate change and the impact of demographic structural changes, such as a shrinking labor force increasing dependency on healthcare systems. BAPC modeling of trends in key G20 countries further supports a long‐term increase in the migraine burden, suggesting that the burden is projected to transition from linear to accelerated growth in the coming decades.

#### 3.2.4 Part4 BAPC Analysis

**FIGURE 19:**
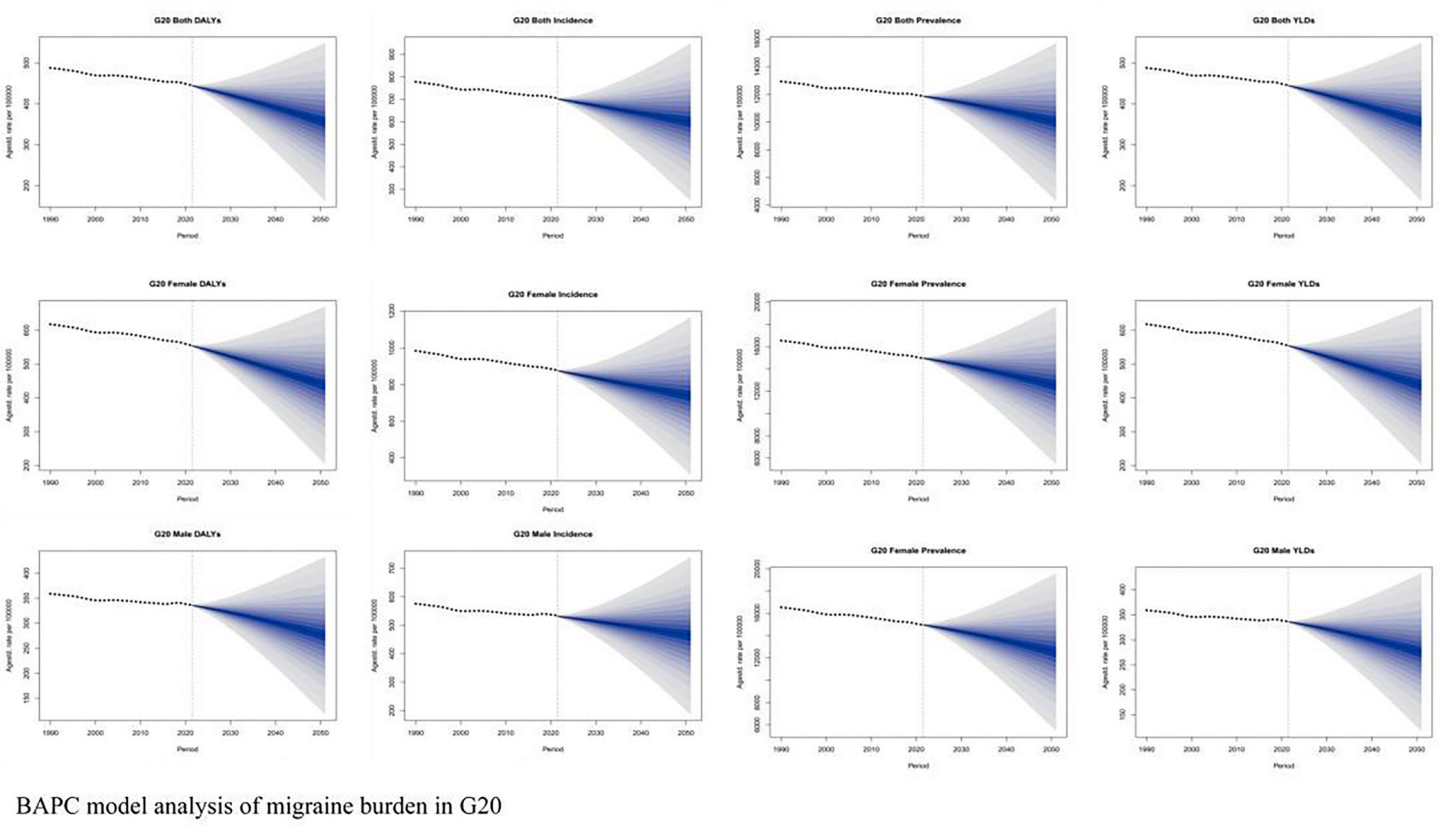
BAPC model analysis of migraine burden in G20.

Our BAPC modeling of trends in key G20 countries provides a sobering long‐term perspective (Figure 19). The analysis suggests that the drivers of the migraine burden are deeply embedded in demographic and generational trends. The dominant age effect confirms that the burden will remain concentrated in the most economically productive years of life. More concerning is the cohort effect, which indicates that successive generations, particularly those born after 1980, may face a progressively higher lifetime risk of migraine. This finding implies that even if period‐specific risk factors were to stabilize, the population‐level burden is likely to continue intensifying for decades as these higher‐risk cohorts age.

Looking towards 2050, our forecasting models, supported by the insights from BAPC analysis, project a transition from the linear growth observed from 1990–2021 to a potential phase of accelerated growth post‐2035. Several external factors, while not formally modeled in this study, could further shape this trajectory. For example, emerging evidence suggests a potential link between rising global temperatures and increased migraine attack frequency, a phenomenon described as climate‐associated attack amplification. Furthermore, challenges in maintaining or improving therapeutic efficacy in the face of evolving disease patterns and potential stalls in therapeutic innovation could also impact the future disability burden. These long‐term trends underscore the urgent need for proactive public health strategies that not only manage existing cases but also address the underlying risk factors affecting younger generations.

## 4 Discussion

This study leveraged three decades of GBD 2021 data to conduct a comprehensive, multidimensional analysis of the migraine burden in China and other G20 nations. By integrating historical trend analysis, decomposition of drivers, and multi‐model forecasting, our research provides a granular view of the evolving, deeply gendered nature of migraine and offers critical evidence for future public health planning. Our findings underscore that migraine is not a static condition but a dynamic public health challenge, shaped by complex interactions between demographic shifts, societal factors, and intrinsic biological vulnerabilities.

Our analysis confirmed a tripartite stratification of the migraine burden. First, the age dimension revealed that the burden is overwhelmingly concentrated in the working‐age population (35–54 years), directly impacting economic productivity and human capital. Second, the gender dimension was profound and universal across the G20, with females consistently bearing a burden approximately twice that of males. This disparity, most pronounced during the reproductive years, aligns with the well‐documented role of hormonal fluctuations, particularly estrogen, in modulating neural excitability (Delaruelle et al. 2018). Third, the disability signature of migraine was unequivocal: YLDs constituted over 98% of DALYs, cementing its status as a leading cause of long‐term disability, not mortality. This triad of characteristics firmly establishes working‐age women as the single group most disproportionately affected by migraine, making them a priority target for intervention. The temporal trends from 1990 to 2021 depicted a concerning “dual‐track” trajectory: A relentless rise in the absolute number of cases, primarily driven by population growth and aging, alongside a concurrent and widening gap in ASRs between sexes. The stagnation of male rates juxtaposed with the steady increase in female rates suggests that factors beyond general demographic changes are at play. This divergence may reflect a combination of structural explanations and measurement issues. Structurally, rising female participation in the workforce, coupled with persistent inequalities in domestic responsibilities, may create a synergistic effect of psychosocial stress. From a measurement perspective, increased health literacy, reduced stigma, and improved diagnostic practices over the past decades may have led to better case ascertainment, particularly among women, who are more likely to seek healthcare.

While previous GBD‐based studies have outlined the global scale of migraine (Li et al. 2023, Y. Yang and Cao 2023), our study provides several unique contributions. By focusing on the G20, a globally influential policy‐making bloc, and conducting a direct comparative analysis with a deep dive into China, we offer insights relevant to both high‐income and major emerging economies. Our decomposition analysis quantified the distinct drivers of burden—aging in high‐income countries versus population growth in middle‐income nations—providing a nuanced understanding that generic global analyses may miss. Furthermore, the integration and validation of forecasting models to project the widening gender gap through 2050 provides a crucial, forward‐looking perspective that is essential for proactive health system planning. The BAPC model's finding of a rising lifetime risk in younger cohorts is particularly alarming, suggesting that the public health challenge of migraine is set to intensify for generations to come.

The escalating and increasingly gendered trajectory of the migraine burden demands urgent, concrete, and sex‐responsive policy action. Broad calls for “targeted interventions” are insufficient; specific recommendations are warranted. Given the concentration of burden among working‐age women, employers and policymakers in G20 nations should develop and implement supportive workplace accommodations, such as flexible work arrangements and educational programs to reduce stigma. For populous, middle‐income G20 countries, strengthening primary care is paramount. This includes training physicians in accurate diagnosis and ensuring access to a full range of evidence‐based treatments, from triptans to newer preventive medications like CGRP monoclonal antibodies and gepants. In addition, public health campaigns should incorporate targeted screening and health education for high‐risk groups, particularly reproductive‐age and perimenopausal women. Finally, the G20 provides a powerful platform to foster international collaboration on standardizing migraine surveillance and sharing best practices.

However, this study has several limitations that must be acknowledged. Our analysis is entirely dependent on the GBD 2021 estimates, which are themselves based on complex modeling of available data; any inaccuracies or biases in the primary data are inherently carried over into our results. Forecasting is also inherently uncertain. Our projections are based on historical trends and cannot account for future disruptive events, such as major therapeutic breakthroughs. The provided PIs should be interpreted as a plausible range, not a definitive prediction. Furthermore, as an ecological study, our findings cannot be used to infer individual‐level risk or causal relationships. The associations we suggest—for example, between the 2020 anomaly and the COVID‐19 pandemic—are hypotheses that require further investigation. Finally, our exploratory grouping of countries was illustrative and not a formal cluster analysis, and its interpretation should be tempered accordingly.

In conclusion, our analysis reveals that the migraine burden in China and across the G20 is substantial, growing, and disproportionately affecting women, particularly during their most productive years. The projected trajectory indicates that without concerted and targeted action, this gendered public health challenge will significantly worsen in the coming decades. This study provides key evidence that underscores the urgent need for precision prevention strategies, optimized resource allocation, and collaborative global efforts to mitigate the profound impact of migraine on human health and economic prosperity.

## Author Contributions

Software, Data curation, Validation, Formal analysis, Visualization, Writing – original draft, and Writing – review and editing: Rong Yang and Wen Chen. Conceptualization, Investigation, and Methodology: Rong Yang, Wen Chen, Mou Sun, Hao Feng, Bing Chen, Xiaoquan Luo, Zhou Li, Fei Qiao, Hui Tang. Supervision, Project administration, and Resources: Haibo Ren. All authors reviewed the results and approved the final version of the manuscript.

## Funding

The authors have nothing to report.

## Ethics Statement

This study is based on publicly available aggregated data and involves no personally identifiable information; therefore, no ethical approval was required.

## Consent

The authors have nothing to report.

## Conflicts of Interest

The authors declare no conflicts of interest.

## Supporting information




**Supplementary Figure S1**: brb371071‐sup‐0001‐FigureS1.docx

**Supplementary Table S1**: brb371071‐sup‐0002‐TableS1.docx

## Data Availability

The data used in this study were sourced from the Global Burden of Disease Study 2021 database. Specific details regarding data extraction and processing, including complete query parameters and code, are stored in a publicly accessible GitHub repository. https://github.com/RongYang‐chenwen/Migraine‐Disease‐Burden.git.
