# Supplementary material for: Migraine Disease Burden and Trends (1990–2021): A Multidimensional Comparative Analysis of China and Other G20 Countries
Source: Brain Behav. 2025 Dec 7;15(12):e71071. doi: 10.1002/brb3.71071 (PMC12683068; doi:10.1002/brb3.71071)

Fig.S1 Forecast of total prevalent migraine cases in China（2022-2050）（Prevalent cases (in millions) - Distribution by year）


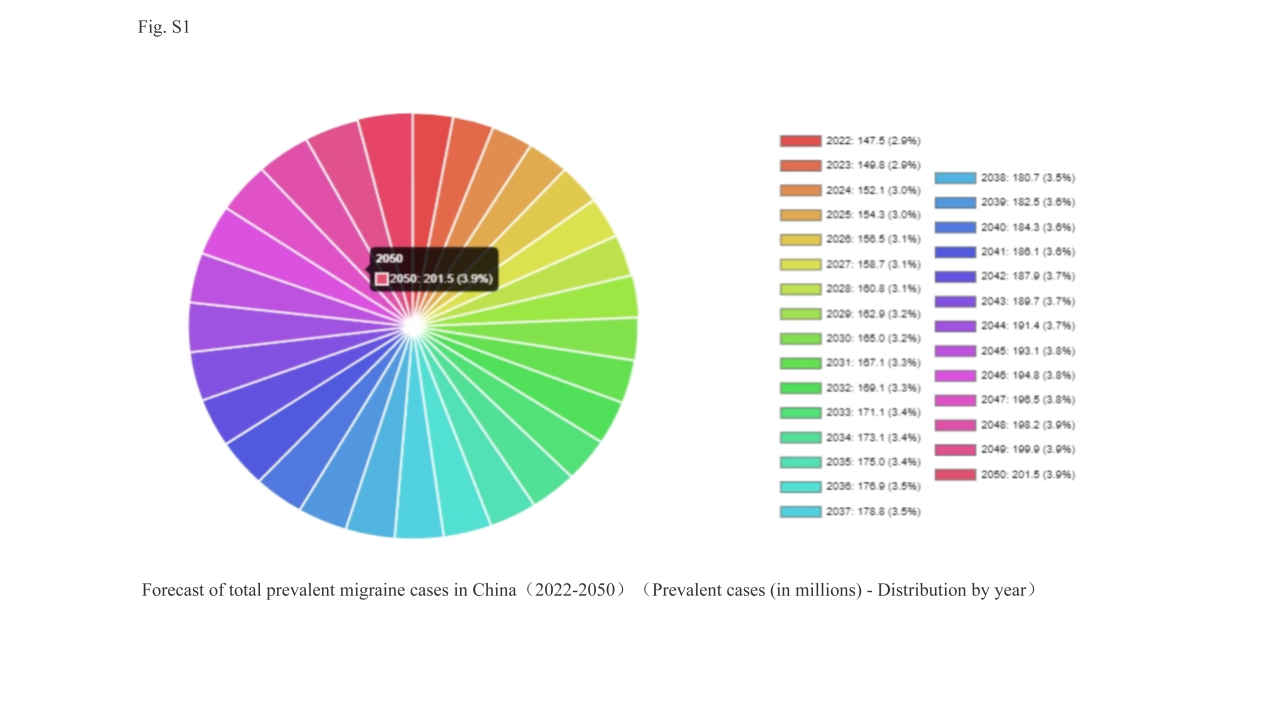

Supplement: Supplementary file 1 — Supplementary Figure S1: brb371071‐sup‐0001‐FigureS1.docx [file BRB3-15-e71071-s002.docx]
