# Supplementary material for: Migraine Disease Burden and Trends (1990–2021): A Multidimensional Comparative Analysis of China and Other G20 Countries
Source: Brain Behav. 2025 Dec 7;15(12):e71071. doi: 10.1002/brb3.71071 (PMC12683068; doi:10.1002/brb3.71071)
Supplement: Supplementary file 2 — Supplementary Table S1: brb371071‐sup‐0002‐TableS1.docx [file BRB3-15-e71071-s001.docx]

**Title: Performance of Time-Series Forecasting Models in an Out-of-Sample Back-Test (Training: 1990–2010, Testing: 2011–2021) for Age-Standardized DALY Rates in China.**

| **Model** | **Sex** | **MAPE (%)** | **RMSE** |
| --- | --- | --- | --- |
| **ARIMA (Autoregressive Integrated Moving Average)** | **Female** | **1.85** | **15.23** |
|  | **Male** | **1.52** | **8.75** |
|  | **Total** | **1.69** | **12.01** |
|  |  |  |  |
| **ES (Exponential Smoothing)** | Female | 2.41 | 19.88 |
|  | Male | 2.15 | 11.54 |
|  | Total | 2.28 | 15.76 |
|  |  |  |  |
| **Benchmark (Random Walk with Drift)** | Female | 3.56 | 28.45 |
|  | Male | 3.11 | 17.61 |
|  | Total | 3.34 | 23.03 |

**Table Footnote:**

MAPE: Mean Absolute Percentage Error. A lower value indicates higher accuracy.

RMSE: Root Mean Squared Error. A lower value indicates higher accuracy. Units are the same as the forecasted variable (age-standardized DALYs per 100,000 population).

The ARIMA model was selected for the main projections due to its consistently lower MAPE and RMSE values across all strata, indicating superior predictive performance in the back-testing validation.
